# Whole-body transcriptome mining for candidate effectors from *Diuraphis noxia*

**DOI:** 10.1186/s12864-022-08712-4

**Published:** 2022-07-07

**Authors:** Vittorio F. Nicolis, N. Francois V. Burger, Anna-Maria Botha

**Affiliations:** Genetics Department, Stellenbosch University, Stellenbosch, 7600 South Africa

**Keywords:** *Diuraphis noxia*, Effectors, DnE’s, *Triticum aestivum*, Aphid-plant interaction, Salivary gland transcriptome mining

## Abstract

**Background:**

Proteins within aphid saliva play a crucial role as the molecular interface between aphids and their host plants. These salivary effectors modulate plant responses to favour aphid feeding and facilitate infestation. The identification of effectors from economically important pest species is central in understanding the molecular events during the aphid-plant interaction. The Russian wheat aphid (*Diuraphis noxia*, Kurdjumov) is one such pest that causes devastating losses to wheat and barley yields worldwide. Despite the severe threat to food security posed by *D. noxia*, the non-model nature of this pest and its host has hindered progress towards understanding this interaction. In this study, in the absence of a salivary gland transcriptome, whole-body transcriptomics data was mined to generate a candidate effector catalogue for *D. noxia*.

**Results:**

Mining the transcriptome identified 725 transcripts encoding putatively secreted proteins amongst which were transcripts specific to *D. noxia*. Six of the seven examined *D. noxia* putative effectors, termed *DnE*’s (*Diuraphis noxia* effectors) exhibited salivary gland-specific expression. A comparative analysis between whole-body *D. noxia* transcriptome data versus the head and body transcriptomes from three other aphid species allowed us to define a catalogue of transcripts putatively upregulated in *D. noxia* head tissue. Five of these were selected for RT-qPCR confirmation, and were found to corroborate the differential expression predictions, with a further three confirmed to be highly expressed in *D. noxia* salivary gland tissue.

**Conclusions:**

Determining a putative effector catalogue for *D. noxia* from whole-transcriptome data, particularly the identification of salivary-specific sequences potentially unique to *D. noxia*, provide the basis for future functional characterisation studies to gain further insight into this aphid-plant interaction. Furthermore, due to a lack of publicly available aphid salivary gland transcriptome data, the capacity to use comparative transcriptomics to compile a list of putative effector candidates from whole-body transcriptomics data will further the study of effectors in various aphid species.

**Supplementary Information:**

The online version contains supplementary material available at 10.1186/s12864-022-08712-4.

## Background

*Diuraphis noxia* (Kurdjumov, Russian wheat aphid) is a phloem-feeding pest that causes significant losses to cereal crop yield. While the damage it causes to rye, triticale and oats is mild, it primarily threatens the production of wheat (*Triticum aestivum*) and barley (*Hordeum vulgare*) throughout small grains production areas worldwide, including Australia [1]. Recent predictions indicate that *D. noxia* may spread further, reaching the United Kingdom and New Zealand as climatic conditions become more favourable [2]. Currently, 17 genes (*Dn1*, to *Dn10* along with *Dnx*, *Dny*, *Dn1818*, *Dn2401*, *Dn2414*, *Dn62658* and *Dn100695*) conferring resistance to *D. noxia* have been reported from wild relatives of bread wheat [3], however, the resistance loci have not been mapped or characterised and the identity of these resistance genes, apart from *Dn2401* [4] remains unknown. While *D. noxia*’s fecundity and lifespan are diminished on resistant cultivars, they can continue to feed and reproduce on these hosts, albeit inducing reduced feeding symptoms [5, 6].

The damage symptoms caused by *D. noxia* feeding are unique (chlorotic streaking and leaf rolling) and while the precise mechanism leading to damage of the host plant requires elucidation, it is clear that feeding by *D. noxia* on susceptible hosts dramatically affects the photosynthetic machinery and capacity of the host plant [7]. Additionally, changes in chlorophyll fluorescence and the efficiency of photosystem II have been observed following *D. noxia* infestation [8], processes in the host plant physiology which have been suggested to take place due to aphid-triggered changes in the carbohydrate source-sink relationship [9] resulting in a nutritionally enhanced phloem sap [10, 11]. As the molecular interface between an aphid and its host is through the aphid’s saliva, it is believed that salivary proteins are responsible for inducing these source-sink changes within the host plant.

Salivary proteins, which perturb host processes, have been termed aphid effectors to reflect their shared features to phytopathogen effectors [12]. These effectors are translocated into host cells and the apoplast during probing and feeding [13] and are critical for mediating the interactions between the host and the aphid [14]. A challenge encountered with effector prediction is their diversity, making effector function difficult to establish based on similarity to other proteins [15, 16]. Saliva from many aphid species has been analysed and found to be composed of a complex mixture of proteins [17–26] that slightly differ between aphid species.

Advances in genomics and transcriptomics have resulted in a number of putative effectors to be predicted from a wide range of aphid species [12, 15, 20, 26–31]. These “omics” approaches have revealed overlap in the effector repertoire between aphid species with varying host ranges, indicating a general set of effectors required for infestation. Despite these similarities, the activity of similar effectors in different species has been found to occur in a plant-species-specific manner indicating that effectors are determinants of aphid host range [14, 32] and reflect the adaptation of aphid lineages to their host plants [15, 28, 30]. Limited progress has been made functionally characterising these proteins [12–14, 27, 32–40] due to the technical challenges associated with these studies.

As with other aphid species, investigations into the salivary protein profile of *D. noxia* have been made. Early studies [41, 42] indicated that the symptoms elicited by *D. noxia* feeding are induced by protein-containing portions of whole-body extracts and that these effectors may differ between biotypes. The proteinaceous nature of the effectors responsible for the host phenotypic response was confirmed by Mohase and Taiwe [43] and were furthermore shown to be present in the aphid saliva. Salivary proteins from *D. noxia* were specifically studied by Cooper et al. [19, 44] which indicated that the salivary protein profile differed in response to host specificity and the type of damage inflicted on the host. *D. noxia* salivary proteins orthologous to those from *Acyrthosiphon pisum* showed a high level of transcript variation within and between two tested biotypes, possibly indicating genes under positive selection pressure in order to adapt to new host cultivars [45]. These proteins may also assist the aphid to avoid detection by the plant surveillance and defence mechanisms [46] as has been shown to be the case in *D. noxia* biotypes [47]. To extend on this debate, a comprehensive analysis by Nicholson et al. [6] of the salivary proteins from four *D. noxia* biotypes in comparison to an *A. pisum* and *Myzus persicae* salivary gland EST database found that the salivary proteome diverged significantly from that of non-phytotoxic aphid species. The detected *D. noxia* species-specific salivary proteins were suggested to reflect the association between *D. noxia* and its host range. Additionally, variability in the salivary proteome and gut transcriptome between aphid biotypes was again observed, indicating a change in the protein profile related to differential biotype virulence [48, 49]. Along with sequence polymorphisms, differential methylation sites were observed in genes putatively encoding enzymes and proteins from the salivary gland between two *D. noxia* biotypes [48]. Taken together, these studies point to a unique array of salivary effectors responsible for the unusual phenotypic symptoms induced by *D. noxia* feeding and its narrow host range on members of the Poaceae. These species-specific effectors are likely highly divergent from those of generalist aphids, as well as from nonphytotoxic aphids, and would play a critical role in promoting the performance and adaptation of *D. noxia* to specific host plants.

As numerous studies from other species have shown that aphids utilise effectors to modulate host responses, it is likely that *D. noxia* makes use of similar mechanisms. Orthologs of characterised salivary effectors have been identified in *D. noxia* [15], although functional characterisation is still lacking. The non-model status of this pest and the complex allohexaploid nature of its preferred hosts signifies that development of the resources required to understand this interaction will be an incremental process. This study therefore aims to broaden the list of candidate salivary effector proteins in *D. noxia* by mining existing transcriptomics data from whole-body insects for protein sequences with characteristics of extracellularly secreted proteins. These candidates were then compared to tissue-specific expression data of three different aphids to predict a catalogue of transcripts putatively upregulated in *D. noxia* head tissue. Together these sources of information create a more accurate effectoromics dataset and form a basis for future functional characterisation studies. Furthermore, the identification of *D. noxia*-specific effectors may act as potential targets for the development of novel control strategies in the future.

## Results

### Putative *D. noxia* effector set prediction

**Fig. 1:**
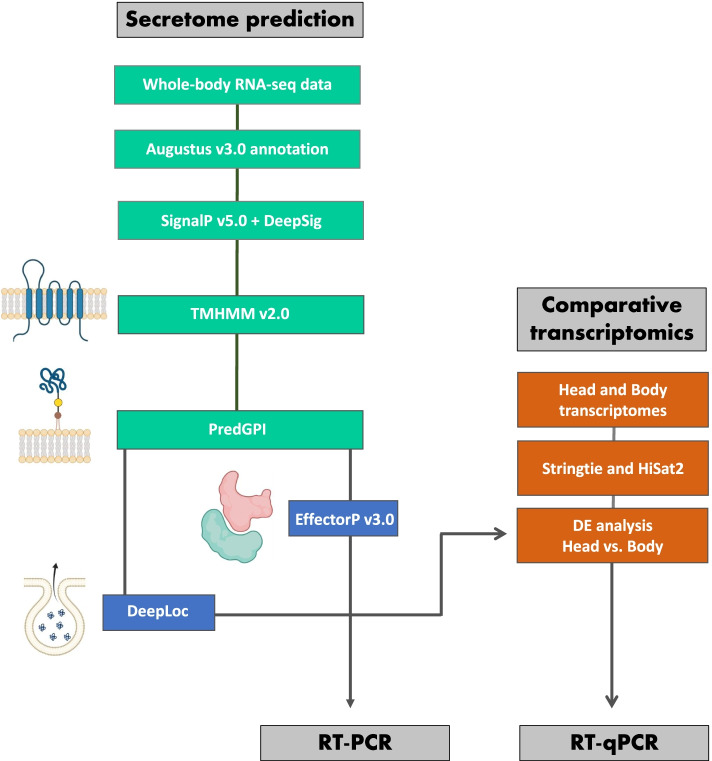
Diagrammatic representation of the methodology used to predict putative effectors from *Diuraphis. noxia* whole-body transcriptome data. RNA-seq data was mined for protein sequences with characteristics of extracellularly secreted proteins. This data was also analysed with EffectorP and combined into a single list, constituting the secretome. Selected sequences were analysed for salivary gland specific expression through RT-PCR. Orthologs of the putatively secreted *D. noxia* sequences were identified in three aphid species. The differential expression of the orthologs between the head versus body transcriptome data of three aphid species was determined to identify transcripts with a Log2-FC>2. This predicted differential expression was confirmed in *D. noxia* head, body and salivary gland tissue through RT-qPCR analysis

Through the combined use of EffectorP v3.0 and a bioinformatics pipeline (Fig. 1) to identify putatively extracellularly secreted proteins, mining whole-aphid transcriptome data resulted in a catalogue of 725 proteins (Additional file 1: Table S1). This constitutes the putative effector repertoire of *D. noxia*. A BLASTp search of the effector set against the NCBI nr database resulted in 703 (out of 725, or 97 %) annotated proteins. Of these putative annotated proteins, 271 (out of 703, or 39 %) were uncharacterised. Additionally, 24 proteins (out of 271, or 9 %) returned no BLAST hit against the NCBI nr database and were considered potentially unique to *D. noxia*, and thus termed DnE’s (*Diuraphis noxia* effectors) A further three proteins, as yet unclassified in Aphididae as putative effectors, were also incorporated into the DnE list. Within the putative effector repertoire of *D. noxia*, 7 proteins were also predicted to be localised to the plastid (Additional file 1: Table S1).

To ascertain the robustness of the proposed methodology, transcripts predicted to encode predicted effectors from whole-body transcriptome data were compared against the *D. noxia* salivary gland transcriptome. Of the 725 putatively secreted proteins from *D. noxia* whole-body transcriptome data, 645 (89 %) returned transcripts matches from the salivary gland transcriptome (Table S1). Of the 27 predicted *DnE*’s, 17 returned significant matches through BLAST against the *D. noxia* salivary gland transcriptome data (Additional file 1: Table S1).

### Tissue-specific expression of putative effectors

**Fig. 2:**
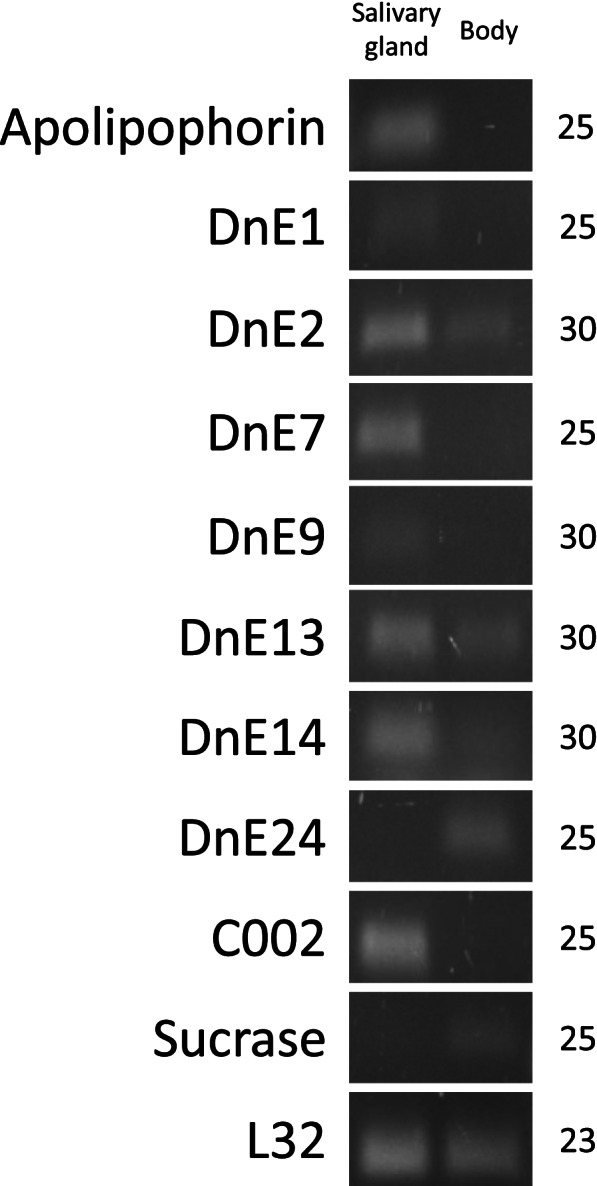
Tissue specific expression analysis of apolipophorin and seven candidate *Diuraphis noxia* effectors (DnE’s). RNA isolated from excised salivary glands or aphid bodies (without heads) was used in reverse transcription semiquantitative PCR with gene specific primers. Preferential salivary gland expression was detected for *apolipophorin* as well as six DnE’s. Expression of *DnC002* and *DnSucrase* were used as controls for salivary glands and body, respectively. Ribosomal gene *L32* was used as an internal control for cDNA input. The numbers on the right indicate PCR cycles at which quantitative differences were observed. The image was compiled from a cropped full-length gel (Additional file 2-Figure S1)

Seven predicted *D. noxia* effector encoding genes (*DnE*’s) that were randomly selected from the list of 27 *DnE*’s (Additional file 1: Table S1), as well as a putative salivary gland specific apolipophorin encoding gene, were investigated for either salivary gland or body tissue-specific expression. RT-PCR analysis found that six *DnE*’s (*DnE1, DnE2, DnE7, DnE9, DnE13* and *DnE14*) and an apolipophorin were preferentially expressed in the salivary glands. Both the salivary gland tissue control (*DnC002*) and body tissue control (*DnSucrase*) [27] were detected in their expected tissues (Fig. 2). The uncropped gel image used to generate Fig. 2 can be found in Additional file 2: Figure S1.

### Comparative transcriptomics and differential expression

**Fig. 3:**
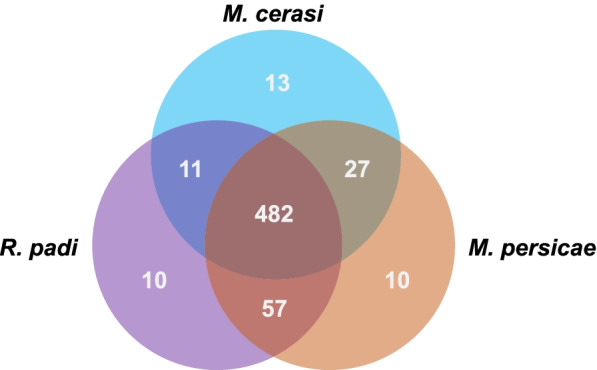
Reciprocal BLASTp matches in three aphid species of *D. noxia* peptides predicted to be extracellularly secreted

Identification of sequences orthologous to *D. noxia* from the three aphid species, resulted in a total of 610 transcripts where at least one species had a reciprocal BLASTp match. Of these, 482 sequences had reciprocal BLASTp matches to *D. noxia* in all three species. Each aphid species had a number of unique reciprocal BLASTp matches with *D. noxia*. *M. cerasi* had the most with 13 unique matches, followed by *M. persicae* and *R. padi* with 10 unique matches each (Fig. 3). Of the 610 *D. noxia* transcripts matched through reciprocal BLASTp searches, 127 had an average Log_2_-FC>2 expression in head tissue from the three aphid species, and 40 had an average Log_2_-FC>2 expression in body tissue from the three aphid species. (Additional file 3: Table S2). *Adhesive plaque matrix protein-like* (TRINITY_DN7331_c0_g1, average Log_2_-FC = 6.06), *prisilkin 39-like* (TRINITY_DN555_c0_g1, average Log_2_-FC = 5.11), *component of gems 1-like* (TRINITY_DN24533_c0_g1, average Log_2-_FC> 502), *Skin secretory protein xP2-like* (TRINITY_DN2183_c0_g1, average Log_2_-FC = 3.83), *vacuolar protein sorting-associated protein TDA6* (TRINITY_DN3334_c0_g3, average Log_2_-FC = 3.10) were found to be the highly expressed transcripts in aphid head data from our study. Five housekeeping genes (*Beta tubulin*, *actin*, *succinate dehydrogenase*, *NADH dehydrogenase* and *ATP synthase*) [50] were included in the differential expression analysis to ascertain if library construction biased the results. None of these housekeeping genes were considered differentially regulated (Log_2_-FC<1, Additional file 3: Table S2).

### RT-qPCR analysis of head up-regulated transcripts

**Fig. 4:**
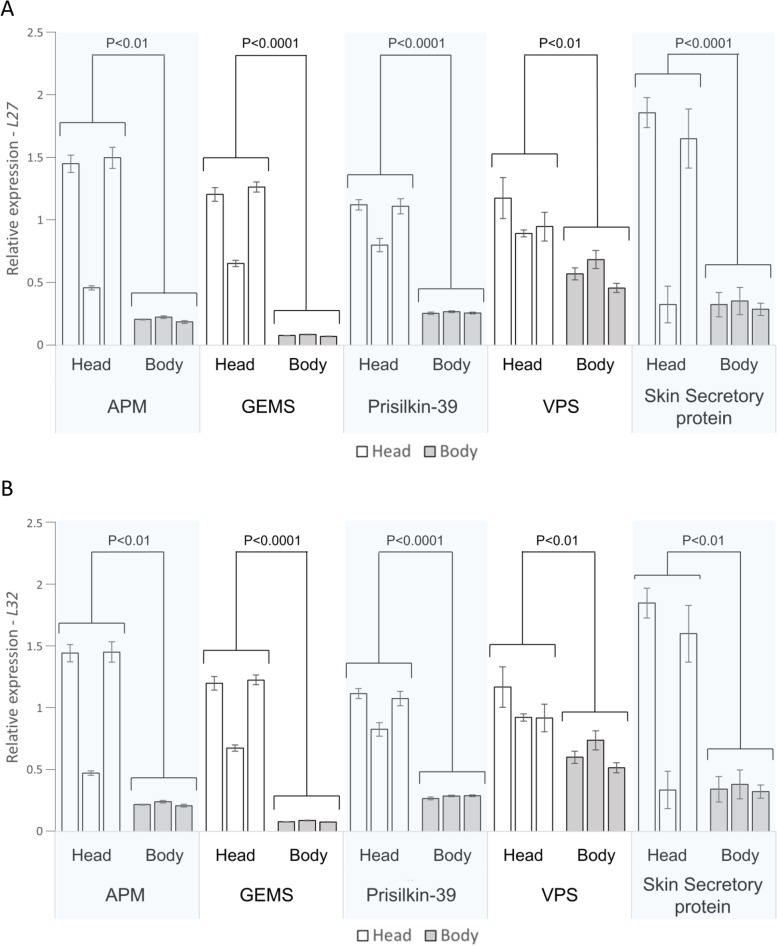
Tissue specific expression analysis in *D. noxia*, through RT-qPCR, of five transcripts predicted to be highly expressed (average Log_2_-FC>3) in head tissue through a comparative transcriptomics approach. All five genes were confirmed to have significantly higher expression in heads compared to body tissue (Student’s t-test, *P*<0.01) when normalised to (**A**) *L27* or (**B**) *L32*. APM = *Adhesive plaque matrix protein-like*, GEMS = Component of Gems protein 1-like, Prisilkin-39 = *Prisilkin 39-like*, VPS = *Vacuolar Protein Sorting-associated Protein TDA6*, Skin secretory = *Skin Secretory Protein xP2-like*. Each bar represents a biological repeat (*n*=3)

**Fig. 5:**
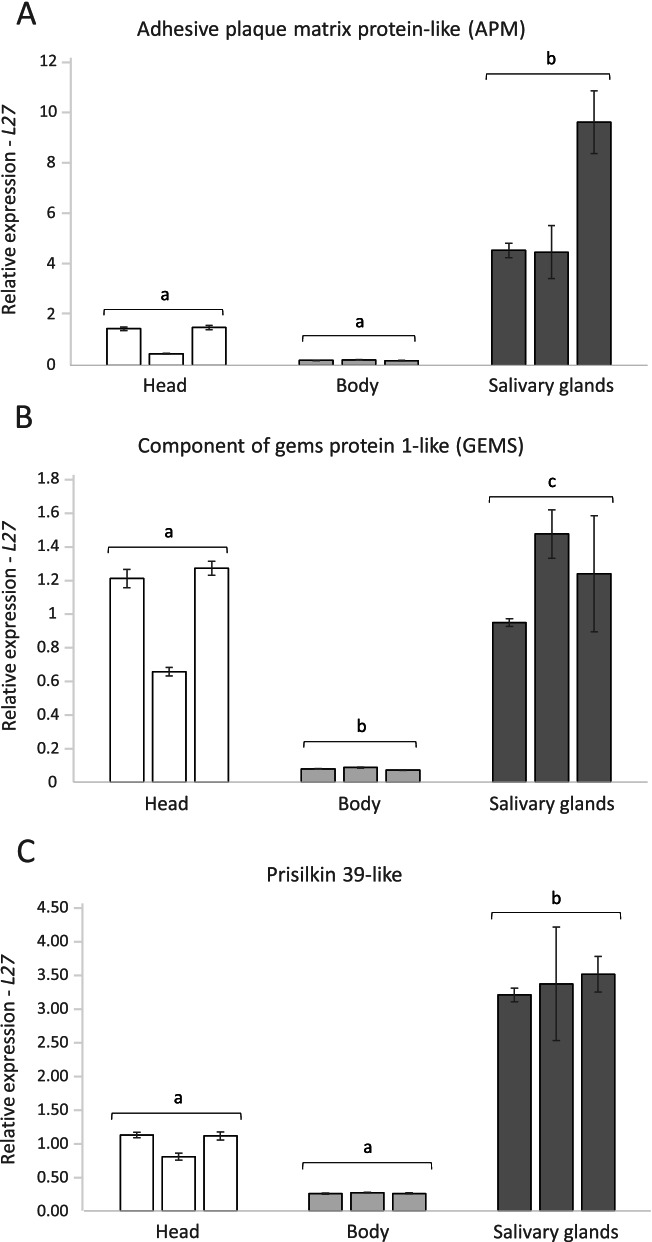
RT-qPCR tissue specific expression analysis in *D. noxia* of (**A**) *Adhesive plaque matrix protein-like*, (**B**) *Component of Gems protein 1-like* and (**C**) *Prisilkin 39-like*, the three most highly expressed head transcripts. All three genes of interest showed significant (ANOVA, *n*=3, *P*<0.05, Tukey's HSD test) upregulation in the salivary glands compared to both head and body tissue, indicating they are predominantly expressed in the salivary glands. Each bar represents a biological repeat (*n*=3)

RT-qPCR validation of the comparative transcriptomics data was performed on five highly expressed (average Log_2_-FC>3) head transcripts (as listed in section 3.3). All five investigated genes showed significant upregulation in *D. noxia* heads compared to the bodies (Student’s t test, *n*=3, *P*<0.05) (Fig. 4 and Additional file 4: Table S3). An additional comparison in expression levels of the three most highly expressed head transcripts between head, body and salivary gland tissue showed significant upregulation in the salivary glands for all three genes of interest (ANOVA, *n*=3, *P*<0.05, Tukey's HSD test) (Fig. 5 and Additional file 5: Table S4).

## Discussion

### Prediction of the putative effector repertoire from *D. noxia* transcriptome data

The majority of aphid effectors are considered to be expressed and synthesized in the salivary glands, which are found in the aphid heads. Typically, salivary gland transcriptomics are applied in the search for insect effectors. However, due to the lack of salivary gland tissue-specific expression data for *D. noxia*, existing transcriptomics data from whole-body insects was mined for sequences with characteristics of extracellularly secreted proteins. A similar approach has been applied to insect species where sequence resources are lacking [51, 52], and has resulted in the identification of pertinent effectors for further functional characterisation.

In this study, whole-aphid transcriptome data was analysed with two effector prediction methodologies, which make use of different strategies (Fig. 1). The protein secretion pipeline makes use of stand-alone tools that determine if certain unifying properties of effectors, such as the presence of signal peptides and lack of glycosylphosphatidylinositol (GPI) anchors, are featured in sequences. The plant-pathogenic effector prediction tool EffectorP is based on machine learning, where it is not limited to the static thresholds of stand-alone tools [53], and has previously been utilised for the prediction of cowpea aphid effectors [26]. Together, these tools identified 725 putatively secreted proteins from the *D. noxia* transcriptome, constituting the predicted aphid secretome (Additional file 1: Table S1), amongst which are several *D. noxia*-specific transcripts that may prove critical in driving the *D. noxia*-wheat interaction. Importantly, this approach applied to whole-body transcriptome data, may result in the identification of putatively secreted proteins that may not act as effectors, and their role will need to be established through functional characterisation studies. While a large number of the predicted effectors are uncharacterised, several share similarity with well-characterised effectors such as Ap25 (XP_015367766), Armet (XP_015365890), Me23 (XP_015370804), Mp1 (XP_015367231), Mp10 (XP_015366710) and Mp55 (XP_015366710). Additionally, members of protein families found to be highly represented in *A. pisum* saliva, such as Angiotensin-converting enzyme-like (ACE) and Aminopeptidase-N were also predicted from our pipeline [15]. The simultaneous knockdown of two highly expressed salivary ACE homologs (ACE1 and ACE2) from *A. pisum* resulted in a higher aphid mortality rate, indicating their role in modulating aphid feeding and survival [54]. The identification of these well-characterised effectors in our dataset suggests that mining a whole-body transcriptome in the search for candidate *D. noxia* effectors is a robust approach in the absence of a salivary gland transcriptome.

Using a salivary gland transcriptome dataset from *D. noxia* biotypes SA1 and SAM feeding on multiple host genotypes, we were able to confirm the presence of 89 % (645 of 725) of the putative effectors predicted from whole-body RNA-seq data (Additional file 1: Table S1). This is significant considering the discrepancy of available transcriptome data on the NCBI for Aphididae tissue types. At the time of submission of this manuscript, there were eight salivary gland transcriptomes, 139 head transcriptome datasets and 1 147 whole-body transcriptomes available. Thus, the methodology proposed here would significantly expand the available datasets for effector prediction in the absence of a salivary gland transcriptome.

#### *D-noxia* effector candidates

Within the predicted effector candidate list, several putatively secreted proteins that have not as yet been catalogued as effectors were identified (Additional file 1: Table S1). These putative proteins were termed DnE’s (*D. noxia* effectors) and are of unknown function. Members of species-specific proteins, termed pioneer effectors, have been identified from all aphid species studied to date, apart from *D. noxia*. Whereas effectors that are conserved across aphid species form part of a “core” effector set, and likely function in general infestation strategies [28], pioneer effectors are predicted to contribute to aphid species-specific infestation strategies. The pioneer effector candidates identified in this study are likely proteins that determine host range and allow *D. noxia* to specifically infest cereals. Furthermore, it is proposed that they may be responsible for inducing the unusual phenotypic changes in the host plant, characteristic of *D. noxia* feeding. While 24 of the identified candidates are *D. noxia* specific, three of the DnE candidates are not. DnE1 shares weak similarity with an uncharacterised protein from *Angomonas deanei*, an endosymbiont-bearing trypanosomatid parasite of insects [55]. Two of the DnE candidates, DnE2 and DnE6, are limited to the Aphididae where they are categorised as uncharacterised proteins. These orthologous proteins could indicate aphid-genera evolution and for that reason are included within the DnE list for future functional characterisation. Of the 27 predicted DnE’s, 17 were detected within the *D. noxia* salivary gland transcriptome data (Additional file 1: Table S1). Although effectors are considered to be primarily expressed from the salivary glands, examples of effectors originating from non-salivary gland sources, such as GroEL and GroES have been confirmed [39, 56], and may suggested the possibility that some DnE’s may be non-salivary gland derived putative effectors, although this requires further validation.

### Chloroplast-targeted putative effectors

Within the putative secretome, seven proteins were predicted to be localised to the plastid (Additional file 1: Table S1). Putative secretory proteins incorporating plastid transit peptides have been reported from other hemipterans [52] although their function has yet to be elucidated. Targeting effectors to important host organelles, such as the chloroplast, is an emerging strategy recognised in bacterial, fungal and oomycete pathogens [57–59]. Pathogens with effectors that target chloroplasts make use of plant subcellular peptide mimics to induce protein translocation to these organelles where they suppress chloroplast function and inhibit plant defences. The host chloroplast plays a critical role in photosynthesis, signalling synthesising major plant hormones, and have been found to actively contribute to the host defence response [60]. Considering that *D. noxia* is a phytotoxic aphid, causing a rapid breakdown of chloroplasts during feeding [8] through a yet unknown mechanism, it is intriguing that putative extracellularly secreted proteins are directly targeted to the chloroplast.

### Tissue specific expression of selected putative effectors

Seven of the eight examined candidate effector genes were preferentially expressed in aphid salivary gland tissue (Fig. 2), suggesting that their corresponding proteins are produced in the salivary glands and may function as effectors. The salivary gland-specific expression of apolipophorin (Additional file 1: Table S1 - XP_015379574.1) was validated in this study as it has been identified in other proteomic and transcriptomic studies as a secreted salivary protein in several aphid species, including *D. noxia* [6, 22–24, 28, 29, 61–63]. While apolipophorins are traditionally linked with lipid transport, they have also been implicated in insect immunity against microbial organisms [64]). Lipids and fatty acids serve as modulators of host plant signal transduction pathways that regulate the host immune response [23, 61, 65, 66]. Salivary apolipophorins secreted into the host during feeding may bind to these signal molecules and interfere with the plant immune response [24]. All predicted DnE’s, apart from DnE1, DnE2 and DnE6, were found to be potentially unique to *D. noxia*, and are proteins with unknown function and no conserved domains or sites. These represent interesting candidates for further characterisation with regards to species-specific roles during aphid infestation. Furthermore, three of the DnE’s are predicted to be localised to the plastid (Additional file 1: Table S1), including the validated salivary gland-specific DnE1. Further validation and characterisation of these *D. noxia*-specific and plastid-localised effector candidates will be required to determine if they are responsible for inducing the unique phenotypic symptoms induced by *D. noxia* feeding. Salivary gland transcriptomics data corroborated the RT-qPCR data (Fig. 2) in that *Apolipophorin* and the six *DnE*’s preferentially expressed from the salivary glands were detected within the salivary gland transcriptome, whereas body-specific expressed *DnE24* was not detected within the salivary gland transcriptome data.

### Transcripts of putatively secreted proteins upregulated in aphid heads versus bodies

As no previous salivary gland transcriptomic data was available for *D. noxia*, predicting effectors for this aphid pest remains a challenge. Generating a list of transcripts upregulated in the aphid head, and therefore potentially in the salivary glands, would aid the study of the molecular events during the aphid-plant interaction. In order to overcome the lack of a salivary gland transcriptome, a comparative transcriptional analysis using aphid head and body data from *M. persicae*, *M. cerasi* and *R. padi* produced a differentially expressed list of orthologous transcripts upregulated in aphid heads versus aphid bodies (Fig. 3 and Additional file 3: Table S2).

Transcripts coding for putative extracellular proteins were used as the input for the comparative transcriptomic analysis. This resulted in 127 orthologous matches preferentially upregulated in aphid heads (Log_2_-FC>2) and 40 matches upregulated in aphid bodies (Log_2_-FC>2) (Additional file 3: Table S2). Thus, 76 % of the significant results (Log_2_-FC>2) were predicted to be more highly expressed in head tissue versus that of the body. As salivary glands function in expressing extracellular proteins for host modulation [34], the head-centric locality of our predicted extracellular proteins points to the robustness of the predictive methodology.

In order to assess if the comparative transcriptomics approach adequately predicted transcripts upregulated in *D. noxia* heads versus the body, the expression of select transcripts were determined. The transcripts selected for this purpose varied in their differential expression levels between the three aphid species compared, ranging from a Log_2_-FC of 6.06 to a Log_2_-FC of 3.1 (Additional file 3: Table S2). All sequences of interest showed significant upregulation (*P*<0.01) in *D. noxia* heads compared to *D. noxia* bodies (Fig. 4 and Additional file 4: Table S3). These results indicate that the use of a comparative approach, from related aphids, is a suitable approach to predict the upregulation of orthologous sequences in *D. noxia* heads. Additionally, they corroborate the predictions made in the comparative transcriptomics analysis and can therefore be used to mine for putative effectors in *D. noxia*, based on high head expression values. As the aphid head contains non-salivary gland tissues, it is likely that the list of head up-regulated genes contains transcripts expressed in organs other than the salivary glands. To assess the sensitivity of the comparative transcriptomics approach in inferring salivary gland-specific transcripts from head up-regulated data, RT-qPCRs were performed on *D. noxia* salivary gland tissue. Three of the five examined transcripts, confirmed to have the highest expression in *D. noxia* heads (Fig. 4), were selected for expression analysis in excised salivary gland tissue. All three genes were significantly upregulated in *D. noxia* salivary glands (Fig. 5 and Additional file 5: Table S4) compared to the other tissues, indicating they are predominantly expressed in the salivary glands. This indicates that a comparative transcriptomics approach, using head and body tissue from related aphid species, to identify highly-upregulated head transcripts can be used to infer preferential salivary gland expression.

While the skin secretory and adhesive plaque matrix proteins have been identified as upregulated in the salivary gland transcriptome of *A. pisum* [15], no aphid-specific expression information is available for the remaining proteins with regards to plant-aphid interactions. However, prisilkin-39 has been shown to tightly bind chitin in multiple organisms [67]. Chitin is well recognised as a pathogen-associated molecular pattern (PAMP) [68], and with its preferential expression in the salivary gland, prisilkin 39-like may serve to sequester chitin oligomers from aphid saliva to suppress PAMP-triggered defence responses.

The results from this study establish that in the absence of a *D. noxia* salivary gland transcriptome, a comparative analysis with closely related species’ RNA-seq datasets (head and body) has allowed for the identification of transcripts preferentially expressed in the salivary glands (Fig. 5). This approach allows for the identification of putative effectors from other aphid species lacking a salivary gland transcriptome, which can be laborious to generate, given the small size of aphids and their salivary glands.

## Conclusion

In summary, this study generated a catalogue of putative *D. noxia* effectors by combining a heuristic *in silico* pipeline approach and a comparative head vs body transcriptome analysis. The identification of transcripts highly upregulated in *D. noxia* head tissue as well as transcripts confirmed to be preferentially expressed in the salivary glands provides the foundation for future characterisation studies where salivary expression must be linked with effector function. Due to the unique phenotypic symptoms induced by *D. noxia* feeding [7] as well as its narrow host range on members of the Poaceae [69], the *D. noxia* specific effectors (DnE’s) identified in this study and shown to be salivary gland specific, are of particular interest. How these pioneer effectors interact with the host plant to modulate its defence responses still needs to be investigated. This study forms the basis for future characterisation of *D. noxia* effectors and depending on their requirement for aphid infestation, may provide novel targets for novel control strategies.

## Materials and Methods

### Predicting putatively secreted proteins from transcriptome data

*De novo* assembled transcripts, obtained through the Trinity pipeline [70], obtained from whole body RNA-seq data of *D. noxia* biotypes SA1 and SAM (NCBI GSE143502) were annotated through use of Augustus v2.5.5 [71]. Two effector prediction methodologies were then applied to the full-length protein sequences obtained from the Augustus annotated transcript set. In the first methods, the presence of signal peptides in the amino acid sequence set were predicted using SignalP 5.0 [72] and DeepSig [73] and all sequences lacking a signal peptide were discarded from further analysis. Sequences that contain more than one transmembrane helix, or a transmembrane helix outside of the N-terminal region, [Predicted by TMHMM 2.0 [74]] or GPI-anchors [Predicted by PredGPI [75]] were removed from further analysis. Finally, proteins that were predicted to be extracellularly secreted [Predicted by DeepLoc 1.0 [76]] were retained as the extracellularly secreted set. In the second method, sequences with a signal peptide (predicted secretome) were analysed with the fungal effector predictor, EffectorP v3.0 [77]. The predicted extracellularly secreted sequences from both pipelines (Fig. 1) were collated into a single non-redundant list and defined as a putative set of effectors. These amino acid sequences were used as queries in a BLASTp search against the NCBI nr database (E-value <10^-5^) and candidates not yet identified as effectors, including several *D. noxia* specific transcripts, were termed *DnE*’s (*D. noxia* effectors). Additionally, a *D. noxia* salivary gland transcriptome (*n*=200 salivary glands from *D. noxia* biotypes SA1 and SAM feeding on various wheat genotypes) was also generated. Salivary glands were dissected in a solution of Phosphate buffered saline (PBS, 0.136 M NaCl, 2.68 mM KCl, 10.1 mM Na2HPO4, 1.76 mM KH2PO4; pH 7.3) followed by transfer to RNAlater (Ambion) until a sufficient number were collected. Immediately prior to RNA extraction, an equal volume of ice-cold PBS was added to the RNAlater to reduce the density of the RNAlater. The tissue was centrifuged at 10 000xg for 10 minutes followed by RNA extraction using a RNeasy mini-kit (Qiagen) as per the manufacturer’s instructions. Extracted RNA was sent to Macrogen (Netherlands) for RNA-seq library preparation using the Illumina TruSeq stranded mRNA kit, for sequencing of the Illumin NovaSeq 6000 instrument. The obtained reads were *de novo* assembled using Trinity [70] to ascertain how many predicted effectors from the salivary gland data matched those predicted from the whole-body transcriptome dataset.

### Tissue-specific expression by semi-quantitative RT-PCR

RNA was isolated from RWA-SA1 headless bodies (*n*=50) and isolated salivary glands (*n*=50). Salivary glands were dissected as indicated previously until a sufficient number were collected. RNA was extracted using a RNeasy mini kit (Qiagen) and cDNA synthesis was performed using a SensiFast^TM^ cDNA synthesis kit (Bioline) according to the manufacturer’s protocol. Gene specific primers for selected DnE’s, *apolipophorin*, *DnC002* and *DnSucrase* as tissue specific controls, as well as the ribosomal *L27* gene as a loading control were designed using Primer 3 (Additional file 6: Table S5). PCR’s were performed using 1X Phusion Hot Start II High-Fidelity PCR Master Mix (ThermoFisher Scientific), 0.3 μM forward and reverse primer, 5 ng cDNA template and water to a final volume of 10 μl. PCR reactions consisted of an initial denaturation 98°C for 1 min, followed by cycling at 98°C for 20 s, annealing at the relevant temperature for each primer pair (Additional file 6: Table S5) for 15 s and an elongation step at 72°C for 20 s, with a final extension of 72°C for 5 min. Reactions were carried out for 20, 25 and 30 cycles, followed by visualisation on a 1 % w/v native agarose gel.

### Comparative head and body transcriptomics from *M. persicae*, *M. cerasi* and *R. padi*

#### RNA-seq data acquisition and trimming

RNA-seq data for head and body tissues from three aphid species (*M. cerasi, M. persicae, R. padi*), was obtained from the SRA database (Additional file 7: Table S6). The obtained reads were then assessed for quality through use of FASTQC [78] and trimmed using Trimmomatic v0.39 [79] to a minimum quality of Q20 over a sliding window of 5 base pairs and minimum sequence length of 40bp. The leading 18bp of all reads were also trimmed to remove adapter content identified by FASTQC.

#### RNA-seq assembly and differential expression analysis

All trimmed reads were aligned to the reference genomes of the various aphids (*M. cerasi*-Myzus.cerasi_genome.v1.1; *M. periscae*–Myzus_persicae_O_v2.0.scaffolds; *R. padi*-R_padi_v2) that were obtained from AphidBase [80] with the HISAT2 program [81]. The obtained SAM files were then converted to BAM files through use of SAMtools [82]. Stringtie [81] was then utilized to assemble and quantify read counts (through use of the prepDE.py script) and differential expression (DE) was then calculated through the use of edgeR [83].

#### Orthologous *D. noxia* sequence assignment

Putatively secreted full-length protein sequences, obtained from the Augustus annotated transcript set of *D. noxia* (NCBI GSE143502), were used to identify orthologous proteins from each aphid species used in the study (Additional file 7: Table S6). Sequences were considered orthologous to each other after a reciprocal BLASTp identified each sequence as the best BLAST match.

#### RT-qPCR analysis of head upregulated transcripts

Five transcripts (*Adhesive plaque matrix protein-like*, *prisilkin 39-like*, *component of gems 1-like*, *Skin secretory protein xP2-like*, *vacuolar protein sorting-associated protein TDA6)* predicted by edgeR to be upregulated in the aphid head compared to the body of three aphid species were selected for validation *in D. noxia* through RT-qPCR expression analysis. Aphid heads (*n*=70), salivary glands (*n*=30) and bodies (*n*=30) of adult apterous *Diuraphis noxia* SA1 biotype aphids feeding on Tugela wheat plants were collected in triplicate. Salivary glands were excised as described above. Heads were separated using a liquid nitrogen-cooled scalpel as previously described [84]. RNA was extracted using a RNeasy mini kit (Qiagen) and cDNA synthesis was performed using a SensiFast^TM^ cDNA synthesis kit (Bioline) according to the manufacturer’s protocol. Primer pairs were designed using Primer3 [85] to produce amplicons of between 102 bp and 130 bp in size. Primers were used in a primerBLAST analysis against the assembled RWA SAM biotype reference genome (GCA_001465515.1) to ensure specificity. The relative expression of the transcripts of interest in aphid heads, salivary glands and aphid bodies was quantified as previously described [86]. A five point, two times serial dilution of a body tissue sample was used to generate quantification standards. All samples and standards were quantified with three technical repeats across three biological repeats along with a no template control for all genes of interest (Additional file 6: Table S5). The ribosomal genes *L27* and *L32* were used as reference genes as in previous studies [86, 87]. A CFX96 Real-Time System (Bio-Rad) was used to perform the PCR analysis. Each reaction included an initial denaturation step at 95 °C for 2 min, followed by 40 cycles of amplification, consisting of a denaturation step at 95 °C for 10 s, an annealing step at the relevant temperature for each primer set (Additional file 6 – Table S5) for 20 s, and an extension step at 72 °C for 20s. A melt curve analysis was also performed for each reaction, to verify the absence of non-specific amplification by increasing the incubation temperature in 5 s intervals, 0.5 °C at a time, from 65 to 95 °C with a plate read at each interval. The relative expression of the transcripts of interest were calculated using Pfaffl's mathematical model [88] for each tissue type. Statistical significance between head and body tissues was determined using a Student’s t-test (*p*<0.05), whereas statistical significance between head, body and salivary gland tissue was determined by a one-way ANOVA followed by a post-hoc Tukey HSD test.

## Supplementary Information

**Additional file 1.**

**Additional file 2.**

**Additional file 3.**

**Additional file 4.**

**Additional file 5.**

**Additional file 6.**

**Additional file 7.**

## Data Availability

The comparative transcriptomics datasets analysed during the current study are available in the NCBI SRA repository, https://www.ncbi.nlm.nih.gov/bioproject?LinkName=sra_bioproject&from_uid=1676321. The whole-body D. noxia transcriptome dataset analysed during the current study are available in the NCBI GEO repository (GSE143502), and the salivary gland transcriptome dataset is available in the NCBI GEO repository (GSE200382).
