# Supplementary figures and images for: Whole-body transcriptome mining for candidate effectors from Diuraphis noxia
Source: BMC Genomics. 2022 Jul 7;23:493. doi: 10.1186/s12864-022-08712-4 (PMC9264610; doi:10.1186/s12864-022-08712-4)

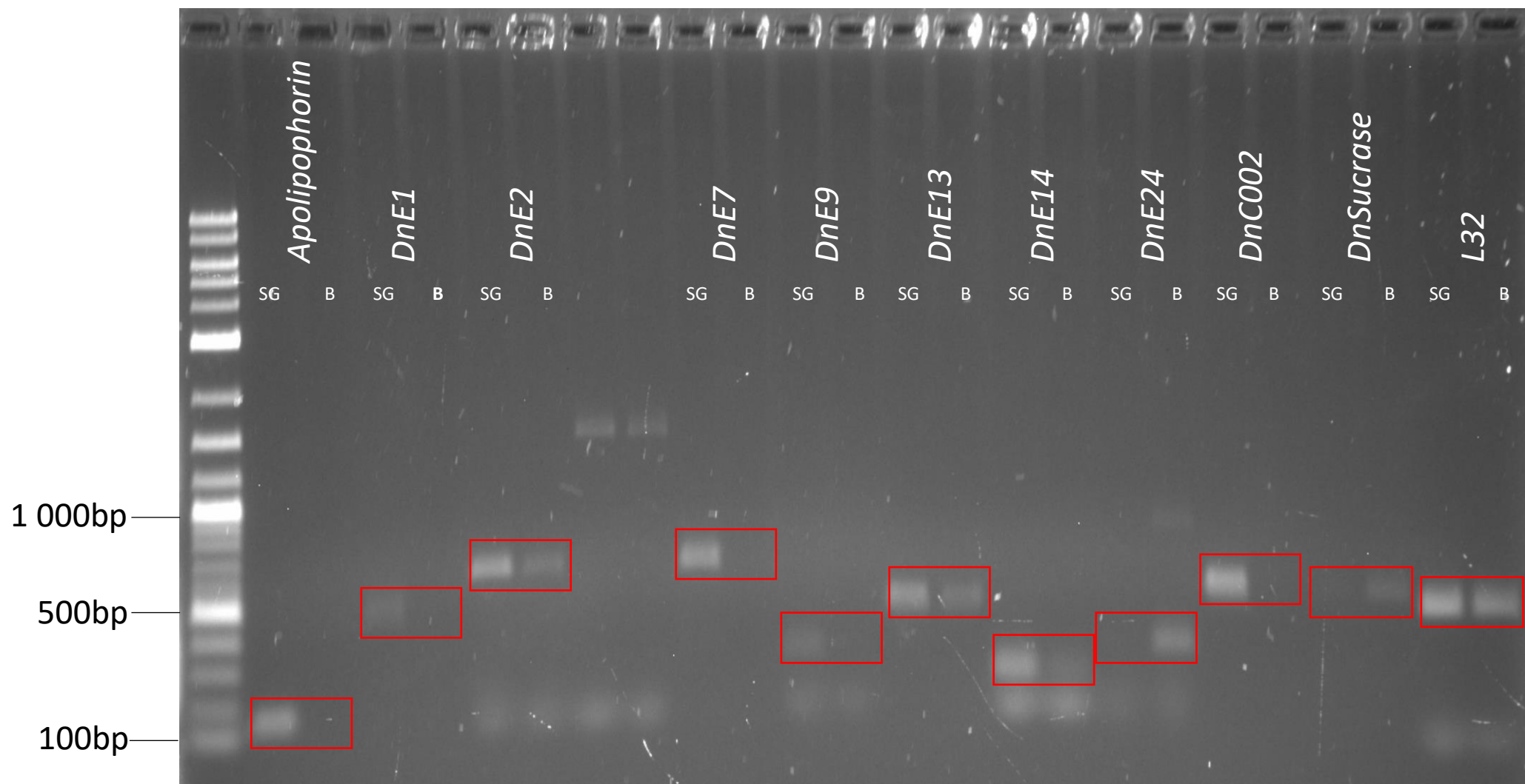

Supplement: Supplementary file 2 — Additional file 2. [file 12864_2022_8712_MOESM2_ESM.pdf]
