# Supplementary material for: Whole-body transcriptome mining for candidate effectors from Diuraphis noxia
Source: BMC Genomics. 2022 Jul 7;23:493. doi: 10.1186/s12864-022-08712-4 (PMC9264610; doi:10.1186/s12864-022-08712-4)
Supplement: Supplementary file 4 — Additional file 4. [file 12864_2022_8712_MOESM4_ESM.pdf]

Additional file 4-Table S3: EdgeR and RT-qPCR fold change values for five genes predicted to be significantly upregulated in aphid head tissue. P-values were calcultated through applying a *t* -test against the average R_{INDIV} values from both head and body tissue obtained from analysing RT-qPCR data through use of the Pfaffl method (Pfaffl, 2001).

Gene	edgeR Log_2FC from comparitive RNA-seq datasets (Head vs. Body)	RT-qPCR estimated fold change **	p-value - L32 ***	p-value - L27 **
Adhesive Plaque Matrix Protein-like	6.06	5.53	5.87E-04	6.37E-04
Component of gems protein 1-like	5.02	13.28	5.92E-06	9.58E-06
Prisilkin-39-like	5.11	3.89	6.28E-07	1.22E-06
Vacuolar Protein Sorting-associated Protein TDA6	3.10	1.76	3.66E-04	1.49E-04
Skin Secretory Protein xP2-like	3.83	3.97	3.76E-06	3.68E-03

**L27 gene used as RT-qPCR normalizer

***L32 gene used as RT-qPCR normalizer
