# Supplementary material for: Whole-body transcriptome mining for candidate effectors from Diuraphis noxia
Source: BMC Genomics. 2022 Jul 7;23:493. doi: 10.1186/s12864-022-08712-4 (PMC9264610; doi:10.1186/s12864-022-08712-4)
Supplement: Supplementary file 6 — Additional file 6. [file 12864_2022_8712_MOESM6_ESM.pdf]

Additional file 6-Table S5: Primer sequences used in RT-qPCR and RT-PCR analysis. RT-qPCR's were performed on five genes that displayed significant upregulation in the the comparative RNA-seq head dataset vs. that of the RNA-seq body dataset. RT-PCR's were performed on seven putative DnE's and an apolipophorin.

Gene	*D. noxia* gene	Forward primer (5'-3')	Reverse primer (5'-3')	Annealing Temp.
Adhesive plaque matrix protein-like	g20974.t2	ACACCTGCTGCAGTTTCTGT	ATGGAGGCTGGTATACGGGT	55
Component of gems protein 1-like	g6654.t2	AACTACCCGTACAACAGCGG	TTGTTGGTAAGCGCCGTACT	55
Prisilkin-39-like	g4715.t2	AATCCCACCACCTGCTTCAG	AACCGTAAGCTTGGCCGTAA	55
Skin secretory protein xP2-like	g11321.t2	CGCCACAACCATATCCGGTA	CACGGGCATGCCGATATAGT	55
Vacuolar protein sorting-associated protein TDA6	g3085.t2	CTACTGCAGCTGTCTTCGCT	GAAACCGCCGCCAAATCTTT	55
L27	g7580.t2	ACCAGCACGATTTTACCAGATTTC	CGTAGCCTGCCCTCGTGTA	55
L32	g3998.t2	CGTCTTCGGACTCTGTTGTCAA	CAAAGTGATCGTTATGACAAACTCAA	54
Apolipophorin	g7743.t2	TGCGTTAGTAGCAGCAACAC	ATGTCTTCCTTCTTTGCCACT	57
DnE1		GAAAACTCCGAACCTGCAGC	TCGGCGTTGTTGATGATTGTT	60
DnE2		CAGCGACGGTAGCGTTACTA	TGGTCGTATCGGTACCTCCA	60
DnE7		TGACGGCGATCTATCAACCG	AAAAGTTTTCGAGCACCGCG	60
DnE9		TGGACACGGCTAGGTTCTTG	ATTCGGCGACTCTGTTGGTG	60
DnE13		TGCTCTTGTCGTTGGTCACA	TCTGTCATTGATGGTGGGCC	60
DnE14		TGGTAGCCACTGTTGTAGTAGC	TGTTTTCAAATCGGCAACAACA	60
DnE24		GGCTCCTCAAGGGACCTGAT	ACACAGTTCGGACATGGCTT	60
DnC002	g23817.t2	AGAGCAGGAAGAAGTGTCCG	CAGTCGGCTTCTCATACGGG	60
Sucrase	g998.t2	AGACTGGTGGCAAACGGAAA	GCGGTGAGATTTCCATGTGC	60
L32-RT-PCR	g3998.t2	AGCCAAAATATCGCCCACAA	ATACGTGCGTTTCCATTGGT	60
